# Ultra-high open-circuit voltage of perovskite solar cells induced by nucleation thermodynamics on rough substrates

**DOI:** 10.1038/srep46141

**Published:** 2017-04-12

**Authors:** Yan Li, Bin Ding, Qian-Qian Chu, Guan-Jun Yang, Mingkui Wang, Chang-Xin Li, Chang-Jiu Li

**Affiliations:** 1 State Key Laboratory for Mechanical Behavior of Materials, School of Materials Science and Engineering, Xi’an Jiaotong University, Xi’an, Shaanxi 710049, P.R. China; 2 Wuhan National Laboratory for Optoelectronics, Huazhong University of Science and Technology, Wuhan, Hubei, 430074, P.R. China

## Abstract

To obtain high performance CH_3_NH_3_PbI_3_ perovskite solar cells, it is highly important to realise a high open-circuit voltage. Calculation results based on a modified diode model have indicated that a low bare ratio *ϕ* of the perovskite film is the most important factor determining the open-circuit voltage, where *ϕ* is defined as the ratio of the projection of the uncovered area of the perovskite film to the apparent area of the total substrate surface. To realise a low *ϕ*, we investigate the nucleation behaviour of crystals on rough substrates. The analysis results predict that, when CH_3_NH_3_PbI_3_ is deposited on conventional transparent conductive oxide substrates such as fluorine-doped tin oxide, preferential heterogeneous nucleation will occur on the concave regions of the substrate; then, depending on the subsequent growth step, full coverage of the perovskite film at both the macroscopic and microscopic scales is realised. As a result, an ultra-high open-circuit voltage, *i.e.*, 1.20 V, can be achieved in devices using the full coverage CH_3_NH_3_PbI_3_ film. The thermodynamics theory of precipitation nucleation should shed light on solution engineering of thin films.

Perovskite solar cells (PSCs) are another promising candidate, after dye-sensitised solar cells, for converting sunlight to electricity^1^,^2^. Many efforts have been made to improve their conversion efficiency^3^,^4^,^5^. A high open-circuit voltage (*V*_oc_) is known to be important for obtaining high conversion efficiency. Previous results suggested that a *V*_oc_ exceeding 1.1 V may have been obtained in CH_3_NH_3_PbI_3_ PSCs by reducing the non-radiative losses^6^,^7^. Recently, Snaith *et al*.^8^ even estimated that the maximum *V*_oc_ is close to 1.3 V for CH_3_NH_3_PbI_3_ PSCs and can be realised by filling the deep traps in the material. The ideal *V*_oc_ is higher than the normally reported value of ~1.0 V^9^,^10^,^11^,^12^; therefore, it is vital to identify the factors determining *V*_oc_ to realise a high *V*_oc_.

The *I*−*V* properties of heterojunction solar cells can be analysed using the diode model^13^,^14^. According to the diode model, when the shunt resistance *R*_sh_ is large enough, *V*_oc_ can be determined from the photo-irradiated constant current (*J*_L_) and reverse saturated current of a PN junction diode (*J*_0_) because 
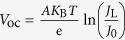
, where A is the ideality factor of a heterojunction, *K*_B_ is the Boltzmann constant, *T* is the absolute temperature, and e is the elementary charge^13^,^15^. However, the shunt resistance *R*_sh_ is not always large enough^16^,^17^,^18^; for example, a Schottky-based CsSnI_3_ solar cell showed a *V*_oc_ of 0.42 V at a low *R*_sh_. Therefore, *V*_oc_ is influenced not only by *J*_L_ and *J*_0_, but also by *R*_sh_. In PSCs with planar configuration, which are more suitable for industrial roll-to-roll processes^19^,^20^,^21^, the perovskite film is sandwiched between the electron transport layer and hole transport layer^22^,^23^,^24^. Because the perovskite film always exhibits incomplete coverage when it is deposited by a one-step solution deposition process^25^,^26^,^27^, both shunting pathways and deleterious effects on charge dissociation, transport and recombination will be introduced in the uncovered areas^28^,^29^,^30^,^31^, strongly degrading the shunt resistance *R*_sh_. Therefore, to achieve a high *V*_oc_ for planar PSCs, it is necessary to clarify the effect of non-full coverage areas on *V*_oc_.

To reduce the shunt resistance *R*_sh_ resulting from the non-full coverage areas, it is necessary to control of the crystallisation process of perovskite films to obtain full coverage on both the macroscopic and microscopic scales. Nucleation is the second step to form the final crystals during the crystallization process, *i.e.* supersaturation, nucleation and growth up. The classical thermodynamics theory of nucleation states that the energy barrier for heterogeneous nucleation (nucleation on the substrate) is lower than that for homogeneous nucleation (nucleation in the solution). Consequently, heterogeneous nucleation on a substrate surface plays an important role in crystallisation of perovskite films^32^,^33^,^34^. In fact, previous results have proved that substrate surface modification (controlling the surface roughness and perovskite/substrate interface energy) is an effective way to enhance the coverage ratio^35^,^36^,^37^,^38^. Docampo *et al*.^35^ obtained coverage ratio of 80% for perovskite films on an indium tin oxide (ITO)/poly(3,4-ethylenedioxythiophene) polystyrene sulfonate (PEDOT:PSS) substrate and 90% on a fluorine-doped tin oxide (FTO)/PEDOT:PSS substrate under the same deposition conditions; the reason for the difference may be that FTO has a rougher surface than ITO. Surface treatment^36^,^37^, such as pre-deposition of 3-aminopropanoic acid on the ZnO substrate, can also greatly increase the perovskite film coverage ratio, mainly by increasing the substrate’s miscibility with perovskite crystals^38^. In addition, the surface properties of the substrate can also affect the microstructure of perovskite films^32^,^39^. However, classical heterogeneous nucleation shows a spherical crown morphology as well as a constant contact angle *θ* at the solution/substrate/nucleus interface, so it requires a uniform flat substrate surface. This is not the case for common substrates, such as FTO and ITO, which always have a rough surface^40^,^41^,^42^. Therefore, it is necessary to investigate the nucleation behaviour on rough substrates to control the crystallisation process of perovskite film.

In this study, the detrimental effect of areas of non-full coverage in perovskite films on the *V*_oc_ value of planar PSCs was first investigated using a modified diode model. To reduce this effect, we further developed the thermodynamics theory of nucleation of perovskite on a rough substrate surface. On the basis of these analyses, full coverage of CH_3_NH_3_PbI_3_ films on rough substrates was experimentally realised. Finally, planar PSCs were assembled and exhibited a maximum *V*_oc_ of 1.20 V owing to a limited shunt path in the solar cells.

## Results and Discussion

### Correlation between areas of non-full coverage of perovskite films and open-circuit voltage for planar configuration PSCs

**Figure 1:**
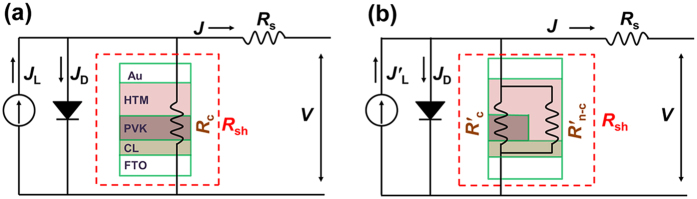
Diode model for full coverage PSCs and non-full coverage PSCs: (**a**) standard diode model with zero bare ratio (*ϕ* = 0) and (**b**) modified diode model with bare ratio *ϕ* (0 < *ϕ* ≤ 100%). *ϕ* is defined as the ratio of the projection of the uncovered area in a perovskite film to the apparent area of the total solar cell. The configuration of both solar cells is FTO/compact layer (CL)/perovskite film (PVK)/hole transport material (HTM)/Au from bottom to top. *J*_L_ is the light induced constant current. *J*_D_ is the diffusion current of the PN junction. *R*_sh_ is the total shunt resistance of the solar cell. *R*_c_ is the shunt resistance when the perovskite film fully covers on the substrate surface. 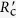 is the shunt resistance in the covered areas. 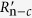 is the shunt resistance in uncovered areas. *R*_s_ is the series resistance.

Herein, we determine the correlation between the area of non-full coverage and the *V*_oc_ value of planar PSCs using the diode model. In a standard diode model (Fig. 1a)^13^, there is only one recombination pathway (*R*_sh_) in the cell. The *I*−*V* characteristic of a heterojunction solar cell are given by


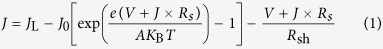


In the open-circuit state (*i.e., J* ≈ 0 mA · cm^2^), *V*_oc_ and *R*_sh_ have the following relationship:


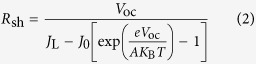


where *J* is the current passed through the external circuit; *V* is the output voltage; *R*_sh_ is the shunt resistance, which results from the defects introduced by the preparation process and the material itself; and *R*_s_ is the series resistance, which consists of the sheet resistance of FTO and the contact resistance of the cell.

If the perovskite film does not fully cover on the substrate (Fig. 1b), it will create another recombination pathway to the cell because of direct contact between the compact layer (CL) and hole transport material (HTM) (see the model of the non-full coverage PSC in Fig. 1b). The bare ratio *ϕ* (0 ≤ *ϕ* ≤ 100%) is introduced to represent the degree of incomplete coverage; it is defined as the ratio of the projection of the area not covered by perovskite film to the apparent area of the total substrate surface. When *ϕ* = 0, the perovskite film fully covers the substrate surface. The standard diode model works in this ideal case, and *R*_sh_ is actually equivalent to *R*_c_, where *R*_c_ is the shunt resistance for a perovskite film fully covering on the substrate surface. When *ϕ* = 100%, *R*_sh_ consists mainly of *R*_n−c_, which is the shunt resistance when there is no perovskite film on the substrate. When 0 < *ϕ* < 100%, the *R*_sh_ value of the non-full coverage solar cell is the parallel of the shunt resistance in the covered areas (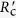) and the shunt resistance in the uncovered areas (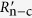). Considering the load connection between shunt resistance, the recombination resistance of the covered areas and uncovered areas can be expressed as


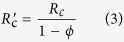



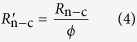


Therefore, the total shunt resistance *R*_sh_ of the modified model is expressed as


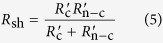


If we substitute equations (3) and (4) into equation (5), then:


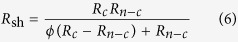


For the non-full coverage PSC, the photo-irradiated constant current 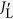 can be expressed as


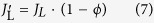


Using equations (6), (7) and (2), the following correlation can be established between the bare ratio *ϕ* and *V*_oc_:


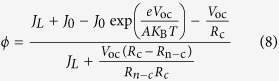


**Figure 2:**
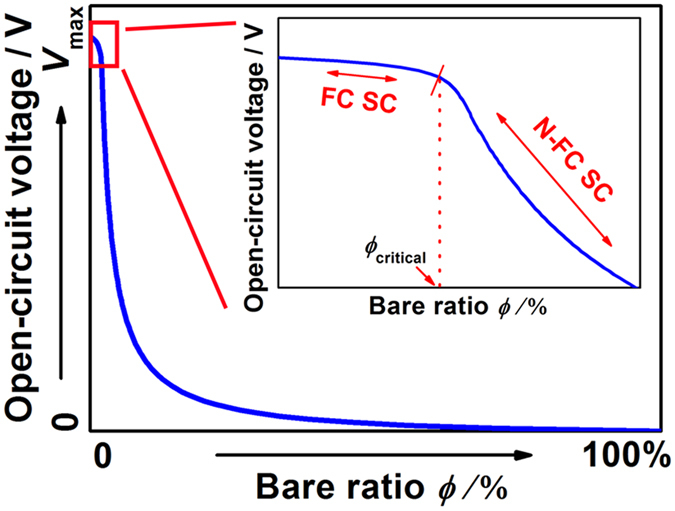
Correlation between bare ratio *ϕ* and *V*_oc_ according to equation (8); the inset shows the part of the curve marked with the red rectangle, and *V*_max_ is the maximum value of *V*_oc_. FC SC: full coverage solar cell. N-FC SC: non-full coverage solar cell.

Figure 2 shows the correlation between the bare ratio *ϕ* and *V*_oc_ according to equation (8). The *V*_oc_ decreases with increasing of the bare ratio *ϕ*, in good agreement with previous results of that showed *V*_oc_ increases with the coverage ratio. The inset shows a magnified view of the curve for small *ϕ*. A critical point (*ϕ*_critical_) clearly appears in the relationship *ϕ* and *V*_oc_. When *ϕ* < *ϕ*_critical_, *V*_oc_ remains almost constant. This case represents a full coverage PSC (FC SC). However, when *ϕ* > *ϕ*_critical_, a remarkable drop in *V*_oc_ is observed with decreasing *ϕ*. This represents a non-full coverage PSC (N-FC SC). On the basis of previous results, *ϕ*_critical_ is usually expected to be extremely small^32^,^38^,^43^,^44^,^45^,^46^,^47^,^48^. Therefore, the significant dependence of *V*_oc_ on *ϕ* for *ϕ* < *ϕ*_critical_ has generally been neglected, and the perovskite film is assumed have full coverage. When *ϕ* is larger than *ϕ*_critical_, it is hard to achieve a high *V*_oc_. To realise a high *V*_oc_, it is essential to realise perovskite films with a *ϕ* lower than *ϕ*_critical_.

### Theory for nucleation on rough surface toward full coverage perovskite film

**Figure 3:**
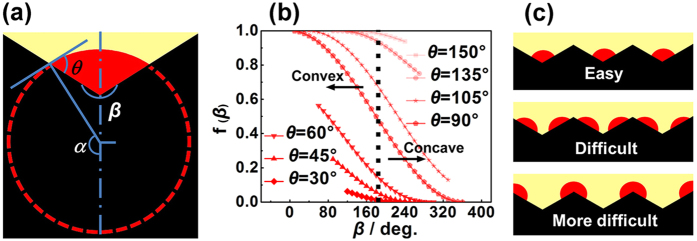
Model of nucleation on a single concavo-convex substrate (**a**), the correlation between the energy barrier Δ*G*_Heter−rough_ and cone angle *β* (**b**), and the sequence of nucleation sites on the rough substrate (**c**).

To realise a full coverage perovskite film by controlling the crystallisation process, it is essential to investigate the nucleation behaviour of crystals on a rough substrate surface. We consider the theory of nucleation on a rough substrate surface as follows. A rough substrate surface always consists of concave and convex regions. To simplify the quantitative description, the concavo-convex substrate surface is assumed to have axial symmetry, *i.e.*, conical concaves existing on the substrate surface (Fig. 3a). As the cone angle *β* increases from 0° to 180° and then 360°, the substrate shape changes from convex to flat to concave (Supplementary Fig. S1). The contact angle *θ* is a function of the energy of the nucleu/substrate/precursor solution interface, which represents the wettability between the perovskite film and the substrate. Table 1 lists the relationship between the cone angle *β* and contact angle *θ*, and the detailed calculation processes are shown in equations (S2)–(S5) in the Supplementary Information. According to the calculation results, the energy barrier (Δ*G*_Heter−rough_) for nucleation on a rough substrate surface can be expressed by equations (9),(10),(11), and the detailed calculation processes are given in equations (S6)–(S21) in the Supplementary Information. Both the cone angle *β* and contact angle *θ* determine Δ*G*_Heter−rough_.


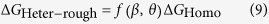



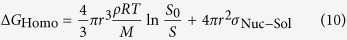



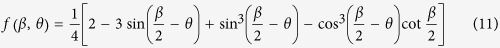


where *σ*_Nuc−Sol_ is the free energy of the nucleus/solution interface.

Figure 3b shows the correlation between the energy barrier (Δ*G*_Heter−rough_) and cone angle *β* according to equation (11).Δ*G*_Heter−rough_ decreases with increasing *β*, and it is always smaller than that for homogeneous nucleation (Δ*G*_Homo_, which corresponds to *β* = 0°) all the time. Therefore, for a constant contact angle *θ*, Δ*G*_Heter−rough_ decreases following the sequence concave < flat < convex, indicating that heterogeneous nuclei form preferentially on sites in order from concave regions to flat regions and then convex regions as shown in Fig. 3c.

**Figure 4:**
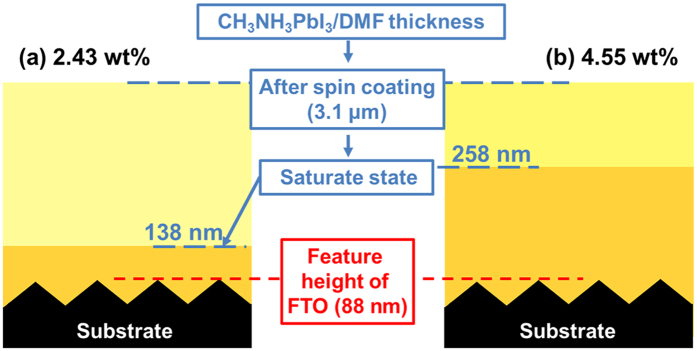
Thickness of CH_3_NH_3_PbI_3_/DMF solutions with low concentration after spin coating and when they are in saturated state for initial solution concentration of: (**a**) 2.43 wt% and (**b**) 4.55 wt%.

To prove this theory, we further performed an experimental validation by depositing CH_3_NH_3_PbI_3_ films with different thicknesses on FTO substrates to identify the nucleation sites. FTO with obvious concavo-convex fluctuation (feature diameter of ~186 nm and feature height of ~88 nm), which will be useful for observation, was selected as an example of a rough substrate. To determine where the nuclei prefer to form during precipitation, the entire substrate, including concave, flat, and convex regions, should be immersed in a supersaturated CH_3_NH_3_PbI_3_/*N,N*-dimethylformamide (DMF) solution during crystallisation process. This ensures the entire substrate has the same active energy for nucleation. In addition, considering the technical challenge of distinguishing the nucleation site *in situ*, the height of a CH_3_NH_3_PbI_3_ film should be smaller than the feature height (88 nm) of the FTO for convenient observation of the nucleation site. Finally, different initial precursors were chosen to obtain the as-prepared perovskite films with mean heights of 18 and 34 nm, and the detailed processes are shown in Fig. 4.

As shown in Fig. 4, first, we determined the thickness of the CH_3_NH_3_PbI_3_/DMF solution left on the substrate after spin coating by measuring the solution weight, which was done by measuring the weight difference between bare FTO and the CH_3_NH_3_PbI_3_/DMF/FTO after spin coating. To reduce the inaccuracy caused by evaporation of the DMF during the measurement, the ambient temperature was kept below 25 °C and the entire weight measurement process reguired less than 1 min. The saturation concentration of the CH_3_NH_3_PbI_3_/DMF solution is ~54.62 wt% at 25 °C. Therefore, when the thickness of the 2.43 wt% initial solution decreases to 138 nm and that of the 4.55 wt% initial solution decreases to 258 nm, they will be in the saturated state. In the saturated state, the thicknesses of the precursor solutions exceed the highest point of the substrate (~88 nm). When the solvent is completely evaporated, the as-prepared CH_3_NH_3_PbI_3_ films show the average heights of 18 and 34 nm, respectively. The height of all of the as-prepared films is below the highest point of the substrate (~88 nm).

**Figure 5:**
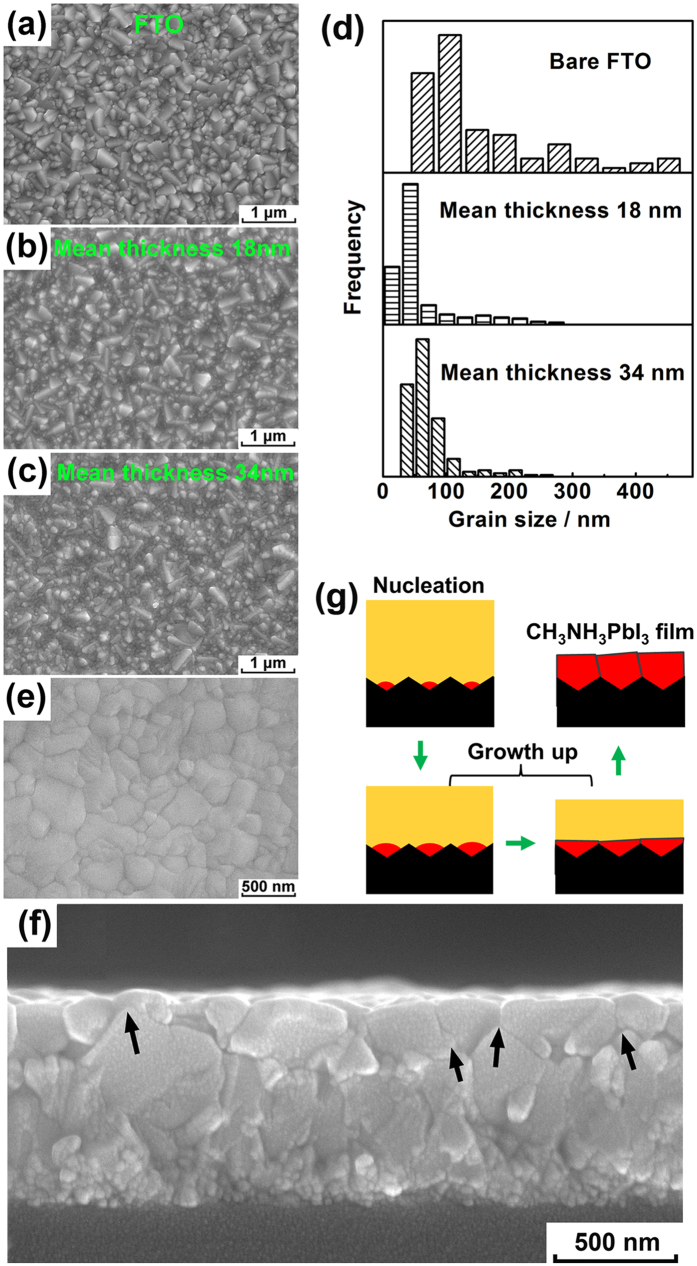
Surface morphologies of (**a**) bare FTO and the CH_3_NH_3_PbI_3_ films deposited on the rough FTO substrate with perovskite layers: having mean thicknesses of (**b**) 18 nm and (**c**) 34 nm. (**d**) Grain sizes of bare FTO and CH_3_NH_3_PbI_3_ crystals deposited on rough FTO substrate. Microstructures of a 240 nm-thick CH_3_NH_3_PbI_3_ film deposited on the FTO substrate surface using a precursor with a concentration of 30 wt%: (**e**) surface morphology and (**f**) cross-sectional view. (**g**) Schematic diagram of formation of full coverage CH_3_NH_3_PbI_3_ film assuming nucleation on concave regions.

The experimental validation results are shown in Supplementary Fig. S7 and S8, Supplementary Table S1, and Fig. 5a–c. The thickness of the perovskite film deposited on the FTO substrate is roughly evaluated by using atomic force microscopy (AFM) images. On basis of the AFM images (Supplementary Fig. S7), the bare FTO shows an average root mean square (RMS) roughness of 45.91 nm. After the precursors with initial concentrations of 2.43 to 4.55 wt% were deposited, the RMS roughness of the substrates decreased from 26.35 to 20.59 nm (Supplementary Fig. S8 and Supplementary Table S1), which illustrates that the perovskite crystals grow up from the concave region of the substrate. Fig. 5a shows the bare FTO substrate. When the perovskite film is about 18 nm thick, the surface of the CH_3_NH_3_PbI_3_/FTO electrode (Fig. 5b) resembles that of the FTO substrate. The CH_3_NH_3_PbI_3_ nanoparticles are concentrated in the valleys between the SnO_2_ grains. As the film thickness increases, the CH_3_NH_3_PbI_3_ nanoparticles appear more clearly between the SnO_2_ grains, as shown in Fig. 5c. In Fig. 5d, the quantitative grain size distribution decreases after CH_3_NH_3_PbI_3_ precipitation. This is because the CH_3_NH_3_PbI_3_ crystals cover the pristine morphology of the FTO substrate. Moreover, the grain size increases slightly as the mean film thickness increases from 18 to 34 nm. This behaviour can be attributed to growth of CH_3_NH_3_PbI_3_ crystals with increasing precursor quantity. These phenomena reveal that all the CH_3_NH_3_PbI_3_ grains form preferentially in concave regions rather than flat or convex region when nucleation begins. This fact directly proves the theory developed in this study; *i.e.*, the Δ*G*_Heter−rough_ value for nucleation is lower in concave regions than in flat or convex regions.

According to the above results, a full coverage CH_3_NH_3_PbI_3_ film can be realised depending on the following three steps, as illustrated in Fig. 5g. First, nuclei appear on the concave regions of the substrate. Second, CH_3_NH_3_PbI_3_ crystals grow upward not only along the out-of-plane direction (perpendicular to the apparent substrate surface), but also along the in-plane direction (parallel to the apparent substrate surface) of the concavo-convex substrate. Third, the CH_3_NH_3_PbI_3_ crystals come to contact with each other in the crosswise direction during growth. Eventually, a full coverage CH_3_NH_3_PbI_3_ film on the substrate can be realised by further film growth and thickening. Fig. 5e shows an example of a ~240 nm-thick CH_3_NH_3_PbI_3_ film prepared by increasing the precursor concentration to 30 wt%. The CH_3_NH_3_PbI_3_ grains nucleated in the concave region and grew upward until they come into contact with each other to form grain boundaries (arrows in Fig. 5f). In addition, the CH_3_NH_3_PbI_3_ film tended to fit itself to the concavo-convex surface of the substrate. The X-ray diffraction (XRD) pattern of the perovskite film shows strong (110), (220), and (310) diffraction peaks and minor (112) and (211) peaks, which are consistent with the tetragonal perovskite phase (Supplementary Fig. S9)^49^.

### Photovoltage of full coverage and non-full coverage PSCs

**Figure 6:**
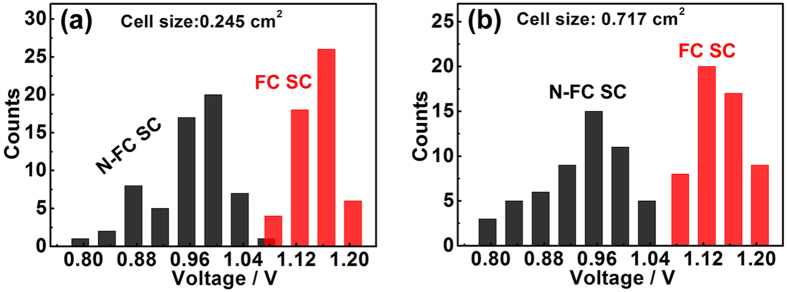
Open-circuit voltages of FC SCs and N-FC SCs: (**a**) with the cell sizes of 0.245 cm^2^ and (**b**) 0.717 cm^2^.

As shown in Fig. 2, a high *V*_oc_ could be achieved by reducing of the bare ratio *ϕ*. Therefore, both the full coverage CH_3_NH_3_PbI_3_ films (*ϕ* < 3%) and the CH_3_NH_3_PbI_3_ films with the bare ratio of 3–6% were assembled into full coverage and non-full coverage PSCs, named as FC SC and N-FC SC separately. Supplementary Fig. S10 shows the cross-sectional views of both solar cells. The photovoltaic performance is shown in Fig. 6. For cell sizes of 0.245 and 0.717 cm^2^, the *V*_oc_ values of the FC SCs range from 1.07 to 1.20 V, and the average value is 1.14 V, whereas those of the N-FC SCs range from 0.80 to 1.07 V, and the average value is 0.98 V. Comprehesive analysis the value of FC SC and N-FC SC, the decreasing of *V*_oc_ is initially small when the bare ratio *ϕ* is low, and then, as *ϕ* increases, there is an obvious drop in *V*_oc_. Furthermore, several PSCs with higher bare ratio *ϕ* (~27%, ~35% and ~67%) were also assembled, and their photovoltaic parameters are listed in Supplementary Table S2. Supplementary Fig. S11 and Supplementary Table S3 show a simple correlation between *ϕ* and *V*_oc_ based on the experiment data presented thus far. Though it is difficult to determine the concrete value of *ϕ*_critical_ on basis of the experimental results in Supplementary Fig. S11, the correlation between *ϕ* and *V*_oc_ is consistent with the prediction in Fig. 2. Note also that the devices show a maximum *V*_oc_ of 1.2 V. This is the highest value reported to date for pure CH_3_NH_3_PbI_3_-based solar cells^50^. The typical current density−voltage (*J*−*V*) curves, taken with both forward and reverse scan, of the perovskite solar cells with *V*_oc_ values of 1.2 and 0.87 V are shown in Supplementary Fig. S12 and Supplementary Table S4, and the detailed photovoltaic parameters of all the PSCs with a *V*_oc_ of 1.2 V are listed in Supplementary Table S5. For the PSCs with a cell size of 0.245 cm^2^, the short circuit density (*J*_sc_) increases slightly with increasing *V*_oc_, as shown in Supplementary Fig. S13, which may result from the decreased bare ratio *ϕ* of the perovskite film. Overall, the FC SCs show a higher conversion efficiency than the N-FC SCs, as shown in Supplementary Fig. S14.

**Figure 7:**
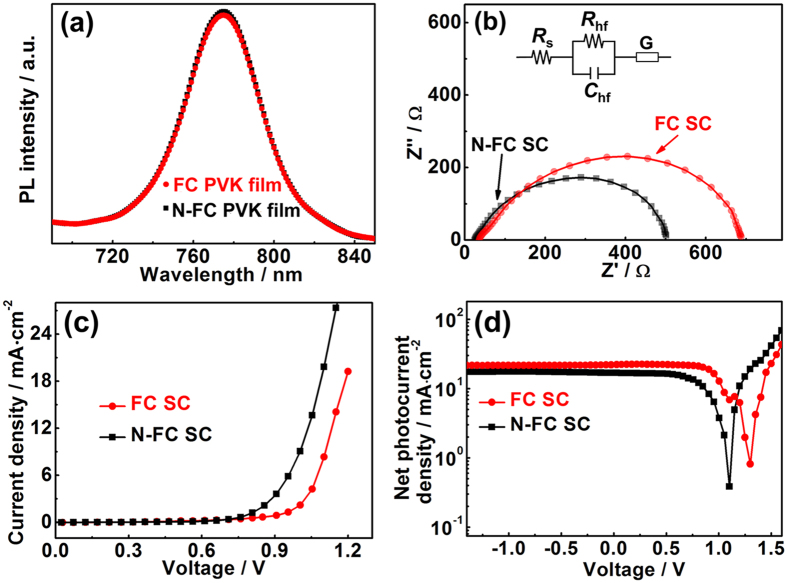
(**a**) Steady-state PL spectra of the FC and N-FC films. Charge transport and recombination processes in FC SCs and N-FC SCs: (**b**) Nyquist plot and (**c**) dark current. (**d**) Net photocurrent of the FC SCs and N-FC SCs. Inset in (**b**) shows the equivalent circuit proposed to fit the EIS data, and the dots and lines in (**b**) represent experimental data and fitting results, respectively.

From Supplementary Fig. S12, the *R*_sh_ values of both solar cells under forward and reverse scanning are estimated from the slope of *J−V* curve at low voltage. The FC SC shows an *R*_sh_ value of 972.88 Ω · cm^2^ (reverse scan), which is larger than the N-FC SC (*R*_sh_ = 384.49 Ω · cm^2^, reverse scan). To comprehensively represent the charge recombination ability of both solar cells, further characterisation are also presented. The photoluminescence (PL) intensities of the cells are comparable (Fig. 7a), which demonstrates that incompletely covered areas in a relatively high coverage state have a negligible effect on the PL intensity. Electrochemical impedance spectroscopy (EIS) results are shown in Fig. 7b. The arc at high frequencies is related to the charge transportation in 2,2′,7,7′-tetrakis(N,N-di-p-methoxyphenylamine) -9,9′-spirobifluorene (spiro-OMeTAD)^51^,^52^. In both spectras, because the conductivity of the hole transport material is large, the arc at high frequencies almost vanishes and cannot be observed. At low frequencies, a Gerischer (G) pattern is clearly identified. The G pattern consists of a straight line with a 45° slope followed by an arc lying below the extension of the straight line^53^. The G resistance (*R*_G_) is the square root of the product of the transport resistance (*R*_tr_) and the recombination resistance (*R*_rec_), so it cannot be separately determined^53^,^54^. The obtained EIS patterns were fitted using the equivalent circuit shown in the inset of Fig. 7b; the FC SC shows a larger *R*_G_ (649 Ω) than the N-FC SC (*R*_G_ = 481 Ω). Although *R*_rec_ cannot be directly estimated from *R*_G_, one can speculate that the FC SC has a larger *R*_G_ value than N-FC SC because of the increased *R*_rec_ resulting from the decreased bare ratio of the perovskite film. The charge recombination mechanism can also be understood in terms of the dark current of both cells (Fig. 7c). The FC SC exhibits a much lower dark current density than the N-FC SC, indicating suppressed charge recombination in the former. This is related to the lower bare ratio of the perovskite film. To further characterise the output voltage capability of voltage of the cells under illumination, the net photocurrent (*i.e.*, the difference between photocurrent and dark current densities versus the voltage, Δ*J*) was tested (Fig. 7d). For a PN junction solar cell, if the dark current is negligible, *J*_sc_ is comparable to *J*_L_, which can be compensated by applying a bias equal to *V*_oc_. However, because the dark current is not negligible, *J*_sc_ will deviate from *J*_L_. There should be a compensating voltage *V*_0_ at which the net photocurrent is zero^55^,^56^. Therefore, *V*_0_ represents the upper limit of *V*_oc_^56^. The FC SC has a higher *V*_0_ (1.31 V) than the N-FC SC (*V*_0_ = 1.12 V), which implies that in principle the FC SC has a higher output voltage capability. In brief, with decreasing bare ratio, the PSCs showed an improved G resistance, a lower dark current and an improved compensation voltage because of the limited recombination circuit, which resulted in high *V*_oc_.

## Conclusion

In summary, a modified diode model is proposed to describe the work principle of planar configuration solar cells, in which a shunt path resulting from direct contact between the electron transport layer and hole transport layer is added to describe PSCs with incomplete film coverage. The calculated results show that when the bare ratio *ϕ* < *ϕ*_critical_, *V*_oc_ remains almost constant, whereas when *ϕ* > *ϕ*_critical_, a slight increase in *ϕ* induces a remarkable drop in *V*_oc_. Furthermore, to decrease the bare area of the perovskite film, we investigated the thermodynamics theory of nucleation on a rough substrate surface by considering the assumed condition of a flat substrate in classical nucleation theory. The theoretical investigation showed that heterogeneous nuclei form preferentially on the concave regions of the substrate. Consequently, full coverage of the perovskite film on both the macroscopic and microscopic scales was realised according to the subsequent growth up. Finally, the full coverage PSCs exhibit an ultra-high *V*_oc_, *i.e.*, 1.20 V for champion cells, which is the highest value reported to date. In brief, the heterogeneous nucleation theory afforded an in-depth understanding of the precipitation behaviour on a rough substrate surface and thus can contribute significantly to the development of high quality perovskite films and high performance PSCs.

## Methods

### Materials

Lead iodide (PbI_2_, purity >99.9%) and methylammonium (ICH_3_NH_3_, purity >99.9%) were purchased from Weihua Solar Co. Ltd.(China). DMF (purity >99.8%) was purchased from Sigma-Aldrich (Germany). Other materials were purchased from Xi’an Polymer Light Technology Corp (China). All the chemicals were used as received unless otherwise specified. Transparent FTO (TEC-15, LOF) conductive glass was employed as a substrate; the substrate were cleaned successively in ultrasonic acetone and alcohol baths and dried under high purity nitrogen gas.

### Perovskite film preparation and characterisation

Precursor solutions of stoichiometric amounts of PbI_2_ and ICH_3_NH_3_ in DMF were prepared at different concentrations by stirring for more than 5 h at 70 °C. Before each perovskite film was prepared, the precursor solution was filtered using a polytetrafluoroethylene filter with a pore size of 0.22 μm. The solution was then dropped on the substrate and spin coating at 4000 rpm for 10 s; it was subsequently dried using a gas pump method^57^. All the processes were performed in atmosphere without inert gas protection. During film preparation, the ambient temperature was kept below 25 °C.

The microstructures of the CH_3_NH_3_PbI_3_ films were examined by field emission scanning electron microscopy (SEM, TESCAN, Czech Republic) and AFM (Innova, America). The crystalline structure of the films was characterised by XRD (SHIMADZU, Japan) using Cu Kα radiation. PL was measured using a steady state spectrophotometer (Fluoromax-4, Horiba Jobin Yvon, France) with an excitation wavelength of 560 nm. The bare ratio of the perovskite films was calculated from the SEM images using the Image J software (Image J2x, 2011), and the detailed statistical process is described in the Supplementary information as Fig. S15 and Fig. S16.

### Planar configuration solar cell preparation and characterization

A thin compact ZnO film was sputtered on the etched FTO substrate (ZnO/FTO substrate) and then annealed at 120 °C for 15 min with a hot plate. Then the perovskite film was deposited on the ZnO/FTO substrate as illustrated in the perovskite film preparation and characterisation section. After the as-prepared perovskite film was annealed at 100 °C for 30 min, the spiro-OMeTAD solution was deposited by spin coating at 3000 rpm for 30 s. The spiro-OMeTAD solution was prepared by dissolving 80 mg of spiro-OMeTAD in 28.5 μl of 4-tert-butylpyridine and 17.5 μl of lithium-bis (trifluoromethanesulfonyl)imide (Li-TFSI) solution (520 mg of Li-TFSI in 1 ml of acetonitrile) in 1 ml chlorobenzene. Finally, an ~80 nm-thick Au layer was thermally deposited on the spiro-OMeTAD film layer.

The photocurrent (*J*−*V*) curves were measured using a Keithley 2400 digital source meter under simulated AM 1.5 sunlight at an irradiance of 100 mW cm^−2^ generated by a solar simulator (Oriel 94023 A, Newport, USA). To measure the dark current, the solar cells were measured without any illumination. The EIS spectra were measured using an electrochemical workstation (IM6, ZAHNER, Germany) under illumination and at an amplitude of 20 mV oover the frequency range from 10^−1^ to 10^6^ Hz.

## Additional Information

**How to cite this article**: Li, Y. *et al*. Ultra-high open-circuit voltage of perovskite solar cells induced by nucleation thermodynamics on rough substrates. *Sci. Rep.* **7**, 46141; doi: 10.1038/srep46141 (2017).

**Publisher's note:** Springer Nature remains neutral with regard to jurisdictional claims in published maps and institutional affiliations.

## Supplementary Material

Supplementary Information

## Figures and Tables

**Table 1:** Cone angle *β* and contact angle *θ* conditions for a stable nucleus to appear on the rough substrate.

If 0° < *β* ≤ 180°, then 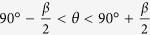
If 180° < *β* < 360°, then 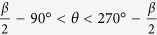
If 0° < *θ* ≤ 90°, then 180° − 2*θ* < *β* < 180° + 2*θ*
If 90° < *θ* < 180°, then 2*θ* − 180° ≤ *β* < 540° − 2*θ*
