# Supplementary material for: Ultra-high open-circuit voltage of perovskite solar cells induced by nucleation thermodynamics on rough substrates
Source: Sci Rep. 2017 Apr 12;7:46141. doi: 10.1038/srep46141 (PMC5388881; doi:10.1038/srep46141)
Supplement: Supplementary Information [file srep46141-s1.doc]

**Electronic Supplementary Information (ESI)**

**Ultra-high open-circuit voltage of perovskite solar cells induced by nucleation thermodynamics on rough substrates**

Yan Li,^a,†^ Bin Ding,^a,†^ Qian-Qian Chu,^a^ Guan-Jun Yang,^a,*^ Mingkui Wang,^b^ Chang-Xin Li,^a^ Chang-Jiu Li^a^

^a^ State Key Laboratory for Mechanical Behavior of Materials, School of Materials Science and Engineering, Xi’an Jiaotong University, Xi’an, Shaanxi 710049, P.R. China

^b^ Wuhan National Laboratory for Optoelectronics, Huazhong University of Science and Technology, Wuhan, Hubei, 430074, P.R. China


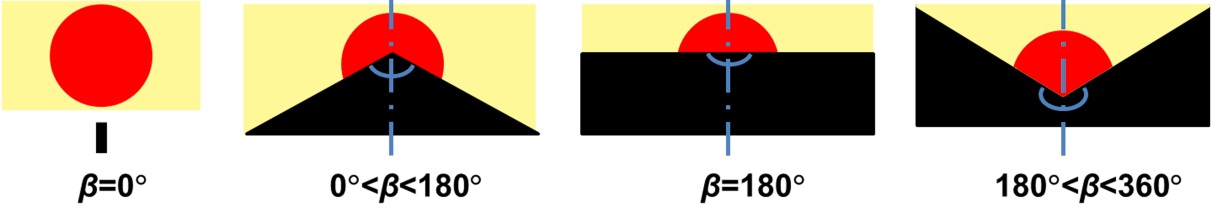


**Figure S1** The substrate shape changes from convex to flat to concave as *β* increases from 0° to 180° and then to 360°. When *β* = 0°, homogeneous nucleation occurs.

**When a stable nucleus appears on a single concavo-convex substrate, the detailed calculated processes of the conditions that the cone angle *β* and contact angle *θ* should satisfy are shown in the following:**

In order to establish the functional relationship between *θ* and *β*, *α* is introduced. *θ*, *β* and *α* has the relationship as following all the time:

$\alpha-\frac{\beta}{2}=90^{\circ}-\theta$ (S1)

It can be seen in Figure S2, for a fix convex substrate *β* (0° < *β* ≤ 180°), at the beginning the nucleus appears in the solvent, which is above the apex of substrate (Figure S2a). This situation is corresponding to the classical homogeneous nucleation. From Figure S2b to S2d the apex of substrate gradually exceeds the nucleus with increase of *α*. Moreover, the contact angle *θ* decreases with the increase of *α*. At last, the nucleus is impaled by the substrate as shown in Figure S2e. Therefore, when *α*_1_ = 0°, there is just nucleus appearing on the substrate, and when *α*_3_ = *β*, the nucleus will be exceeded by the convex substrate as shown in Figure S5d. They are the two boundary conditions to keep a stable nucleus on the fix convex substrate *β* (0°<*β*≤180°). Based on the eqn (S1), the contact angle *θ* should meet the following condition then there will be nucleus appearing on the substrate.

If 0° < *β* ≤ 180°, $90^{\circ}-\frac{\beta}{2}<\theta<90^{\circ}+\frac{\beta}{2}$ (S2)


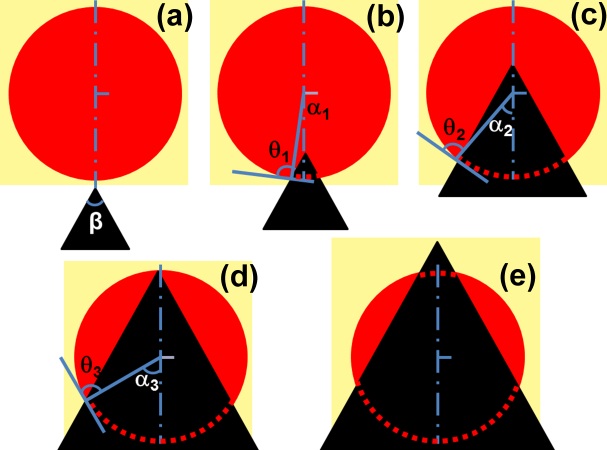


**Figure S2** For a fix convex substrate *β* (0° < *β* ≤ 180°), the nucleus is above the apex of the substrate (a); the apex of the substrate gradually exceeds the nuclei with increasing of *α* (b), (c) and (d); the nucleus is impaled by the substrate (e).

It can be seen in Figure S3, for a fix concave substrate *β* (180° < *β* < 360°), at the beginning the nucleus is below the apex of substrate (Figure S3a), which stands for no nucleus on the substrate. From Figure S3b to S3d, the nucleus gradually appears on the substrate with decrease of *α*. Moreover, the contact angle *θ* increases with the increase of *α*. At last, the nucleus is impaled by the substrate as shown in Figure S3e. Therefore, when *α*_1_=180°, there is just nuclei appearing on the substrate, and when *α*_3_ = *β* - 180°, the nuclei will be exceeded by the concave substrate as shown in Figure S3d. Based on the eqn (S1), the contact angle *θ* should meet the following condition then there will be nucleus appearing on the substrate.

If 180° < *β* < 360°, (S3)


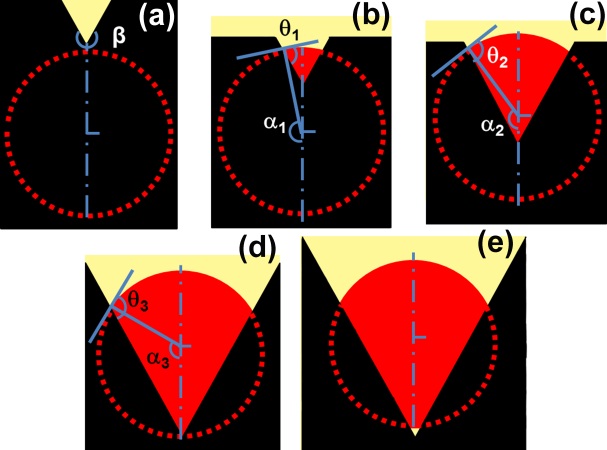


**Figure S3** For a fix convex substrate *β* (180° < *β* < 360°), the nucleus is below the apex of the substrate (a); the apex of substrate gradually exceeds the nucleus with decreasing of *α* (b), (c) and (d); the nucleus is impaled by the substrate (e).

It also can be seen in Figure S4, for a fixed contact angle *θ* (0° < *θ* ≤ 90°), at the beginning the nucleus is below the apex of substrate (Figure S4a), which stands no nucleus at the substrate. From Figure S4b to S4e, the nucleus gradually appears on the substrate. In order to keep the contact angle *θ*, the substrate changes from concave to convex with decreasing of *α*. At last the nucleus is exceeded by the convex substrate in Figure S4f. Therefore, when *α*_1_ = 180°, there is just a nucleus with contact angle *θ* appearing on the substrate, and when *α*_4_ = 180° - 2*θ*, the nuclei will be exceeded by the convex substrate as shown in Fig. S4e. Based on the eqn (S1), the cone angle *β* should meet the following condition then there will be nucleus appearing on the substrate.

If 0° < *θ* ≤ 90°, $180^{\circ}-2\theta<\beta<180^{\circ}+2\theta$ (S4)


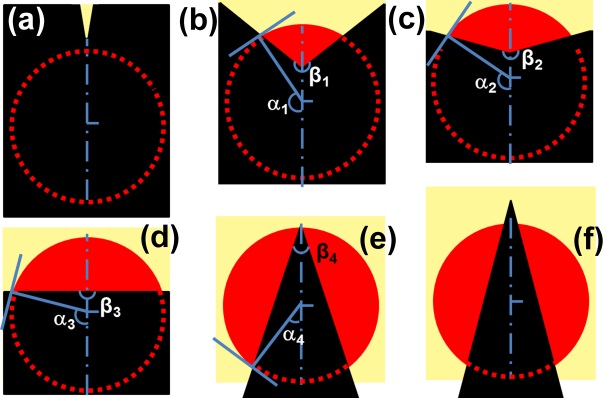


**Figure S4** For a fixed contact angle *θ* (0° < *θ* ≤ 90°), the nucleus is below the apex of the substrate (a); the substrate changed from concave to convex with decreasing of *α* (b), (c) and (d) and (e); the nucleus is impaled by the substrate (f).

Furthermore, for a fixed contact angle *θ* (90° < *θ* < 180°) (Figure S5), at the beginning the nucleus is above the apex of substrate (Figure S5a), which corresponding to homogeneous nucleation in the solution. From Figure S5b to S5e, the apex of substrate gradually exceeds the nucleus. In order to keep the contact angle *θ*, the substrate changed from convex to concave with increasing of *α*. At last the nucleus is exceeded by the convex substrate (Figure S5f). Therefore, when *α*_1_ = 0°, there is just nucleus with contact angle *θ* appearing on the substrate, and when *α*_3_ = 360° - 2*θ*, the nucleus will be exceeded by the convex substrate as shown in Figure S5e. Based on the eqn (S1), the cone angle *β* should meet the following condition then there will be nucleus appear on the substrate.

If 90°< *θ* < 180°, $2\theta-180^{\circ}\leq\beta<540^{\circ}-2\theta$ (S5)


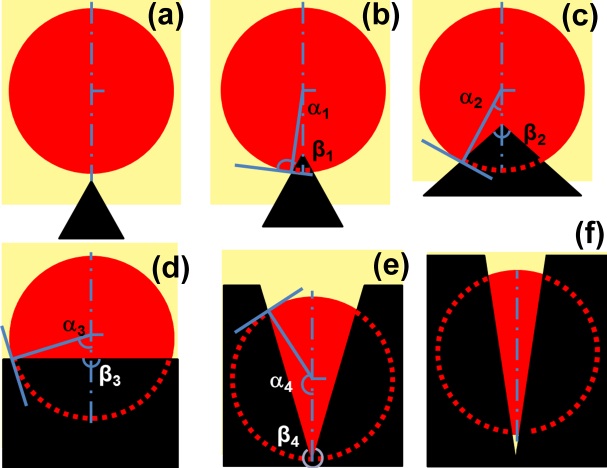


**Figure S5** For a fixed contact angle *θ* (90° < *θ* < 180°), the nucleus is above the apex of the substrate (a); the substrate changed from convex to concave with increasing of the degree *α* (b), (c), (d) and (e); the nucleus is impaled by the substrate (f).

**When there is a nucleus appearing on a single concavo-convex substrate, the bulk free energy and the interface free energy calculation process:**

During nucleation process in solution, the total energy change of the system is the sum of the interfacial energy term and the bulk energy term:

$\Delta G=\Delta G_{Vol}+\Delta G_{Int}$ (S6)

The bulk free energy (Δ*G*_Vol_) is the product of the molar number of the nuclei (*n*) and the change of the solution chemical potential (Δ*μ*), which can be expressed by eqn (S7) – eqn (S9). The interfacial free energy (Δ*G*_Int_) is related to the interface energy of nucleus/substrate (A_Nuc-Sub_*σ*_Nuc-Sub_), nucleus/solution (A_Nuc-Sol_*σ*_Nuc-Sol_) and solution/substrate (A_Sol-Sub_*σ*_Sol-Sub_), which can be expressed by eqn (S10).

$\Delta G_{Vol}=n\Delta\mu$ (S7)

$n=\frac{\rho V}{M}$ (S8)

$\Delta\mu=RT\ln\frac{S_{0}}{S}$ (S9)

$\Delta G_{Int}=A_{Nuc-Sub}\sigma_{Nuc-Sub}+A_{Nuc-Sol}\sigma_{Nuc-Sol}-A_{Sol-Sub}\sigma_{Sol-Sub}$ (S10)

where *S* and *S*_0_ are the concentration of the supersaturation state and saturation state, respectively. A_Nuc-Sub_, A_Nuc-Sol_ and A_Sol-Sub_ are corresponding to the interface areas of the nucleus/substrate, nucleus/solution and solution/substrate, respectively. *σ*_Nuc-Sub_, *σ*_Nuc-Sol_ and *σ*_Sol-Sub_ are the interface free energy of the nucleus/substrate, nucleus/solution and solution/substrate, respectively.

In order to get the bulk free energy (Δ*G*_Vol-Heter-rough_), the mole number (*n*) of the nucleus can be calculated from the total volume of the nuclei (*V*_Heter-rough_). The *V*_Heter-rough_ is composed of the volume of the spherical crown (*V*_1_) and the volume of the cone (*V*_2_) as shown in Figure S6. The calculated process is shown in eqn (S11) – eqn (S13).

$V_{Heter-rough}=V_{1}+V_{2}$ (S11)

$V_{1}=\frac{\pi}{3}r^{3}\left(2+3\cos\alpha-\cos^{3}\alpha\right)$ (S12)

$V_{2}=-\frac{\pi}{3}r^{3}\sin^{3}\alpha\cot\frac{\beta}{2}$ (S13)

**
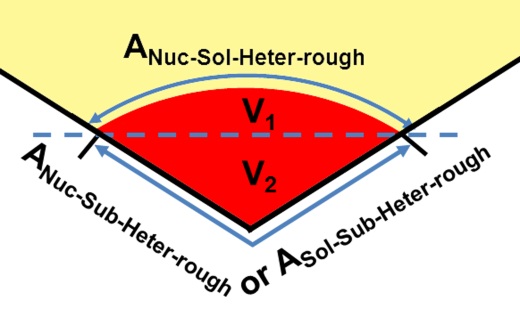
**

**Figure S6** Volume change and surface change during heterogeneous nucleation on rough substrate.

Taking eqn (S8), eqn (S9) and eqn (S11) – eqn (S13) into eqn (S7), the bulk free energy, Δ*G*_Vol-Heter-rough_, which is changed with *β* and *θ*, can be expressed as:

$\Delta G_{Vol-Heter-rough}=\frac{1}{4}\left[2-3\sin\left(\frac{\beta}{2}-\theta\right)+\sin^{3}\left(\frac{\beta}{2}-\theta\right)-\cos^{3}\left(\frac{\beta}{2}-\theta\right)\cot\frac{\beta}{2}\right]\left(\frac{4}{3}\pi r^{3}\frac{\rho RT}{M}\ln\frac{S_{0}}{S}\right)$(S14)

The interface area change of nucleation on a single concavo-convex substrate is also shown as Figure S6. The interface area of nuclei/solution is part of the superficial area of the sphere, and the interface area of the nuclei/substrate or solution/substrate is the lateral area of the circular cone. The calculated results were expressed by eqn (S15) and eqn (S16). At the same time at the contact line of solution/substrate/nuclei, *σ*_Nuc-Sol-Heter-rough_, *σ*_Nuc-Sol-Heter-rough_ and *σ*_Sol-Sub-Heter-rough_ have a fix relationship as shown in eqn (S17).

$A_{Nuc-Sol-Heter-rough}=2\pi r^{2}\left(1-\cos\alpha\right)$ (S15)

$A_{Sol-Sub-Heter-rough}=A_{Nuc-Sub-Heter-rough}=\pi r^{2}\sin^{2}\alpha\frac{1}{\sin\frac{\beta}{2}}$ (S16)

$\sigma_{Sol-Sub-Heter-rough}=\sigma_{Nuc-Sol-Heter-rough}\cos\theta+\sigma_{Nuc-Sub-Heter-rough}$ (S17)

Taking eqn (S15) – eqn (S17) into eqn (S10), the interface free energy, Δ*G*_Int-Heter-rough_, which is also changed with *β* and *θ*, can be expressed as:

$\Delta G_{Int-Heter-rough}=\frac{1}{4}\left[2-3\sin\left(\frac{\beta}{2}-\theta\right)+\sin^{3}\left(\frac{\beta}{2}-\theta\right)-\cos^{3}\left(\frac{\beta}{2}-\theta\right)\cot\frac{\beta}{2}\right]\left(4\pi r^{2}\sigma_{Nuc-Sub-Heter-rough}\right)$(S18)

Therefore, the energy barrier (Δ*G*_Heter-rough_) of nucleation on a rough substrate surface is expressed as:

$\begin{matrix} \Delta G_{Heter-rough}=\frac{1}{4}\left[2-3\sin\left(\frac{\beta}{2}-\theta\right)+\sin^{3}\left(\frac{\beta}{2}-\theta\right)-\cos^{3}\left(\frac{\beta}{2}-\theta\right)\cot\frac{\beta}{2}\right] \\ \left(\frac{4}{3}\pi r^{3}\frac{\rho RT}{M}\ln\frac{S_{0}}{S}+4\pi r^{2}\sigma_{Nuc-Sub-Heter-rough}\right) \end{matrix}$ (S19)

Based on the classical nucleation theory, the energy barrier of homogeneous nucleation (Δ*G*_Homo_) can be expressed as:

$\Delta G_{Homo}=\frac{4}{3}\pi r^{3}\frac{\rho RT}{M}\ln\frac{S_{0}}{S}+4\pi r^{2}\sigma_{Nuc-Sol}$ (S20)

Therefore, the correlation between Δ*G*_Heter-rough_ andΔ*G*_Homo_ can be shown as:

$\Delta G_{Hetre-rough}=f\left(\beta,\theta\right)\Delta G_{Homo}$ (S21)

$f\left(\beta,\theta\right)=\frac{1}{4}\left[2-3\sin\left(\frac{\beta}{2}-\theta\right)+\sin^{3}\left(\frac{\beta}{2}-\theta\right)-\cos^{3}\left(\frac{\beta}{2}-\theta\right)\cot\frac{\beta}{2}\right]$ (S22)


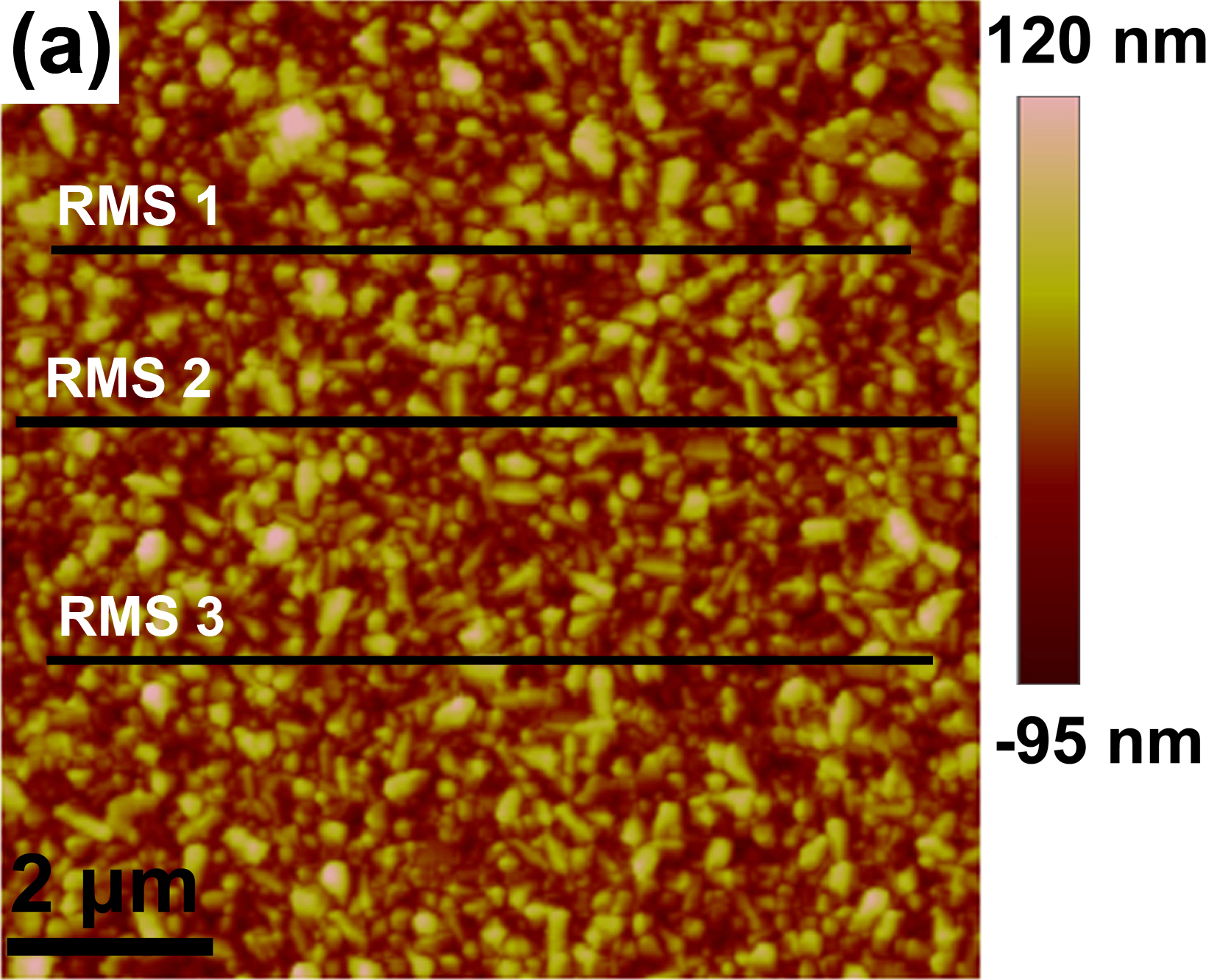

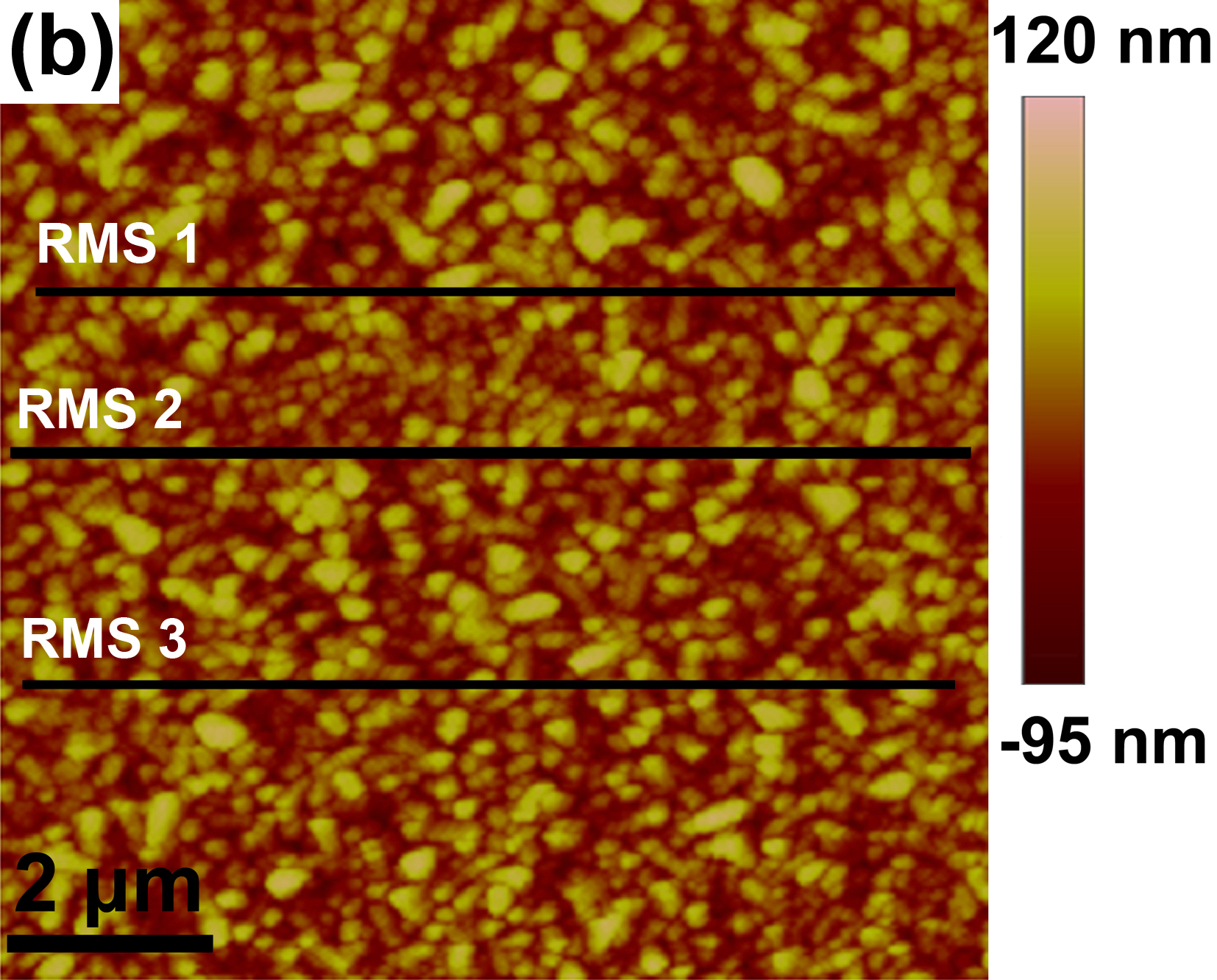


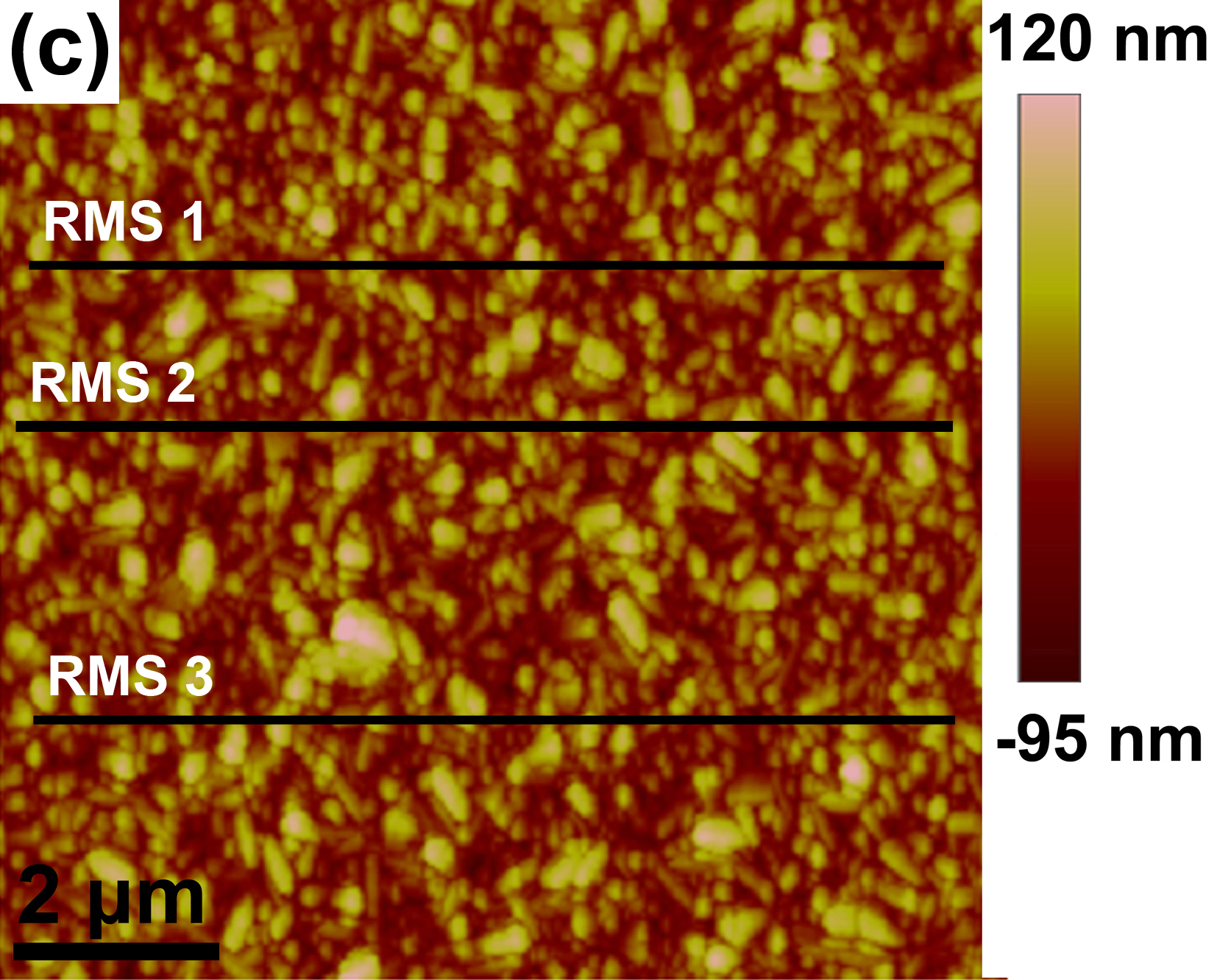


**Figure S7** AFM images of (a) bare FTO and perovskite films deposited on FTO from precursors with initial concentrations of (b) 2.43 wt% and (c) 4.55 wt%.


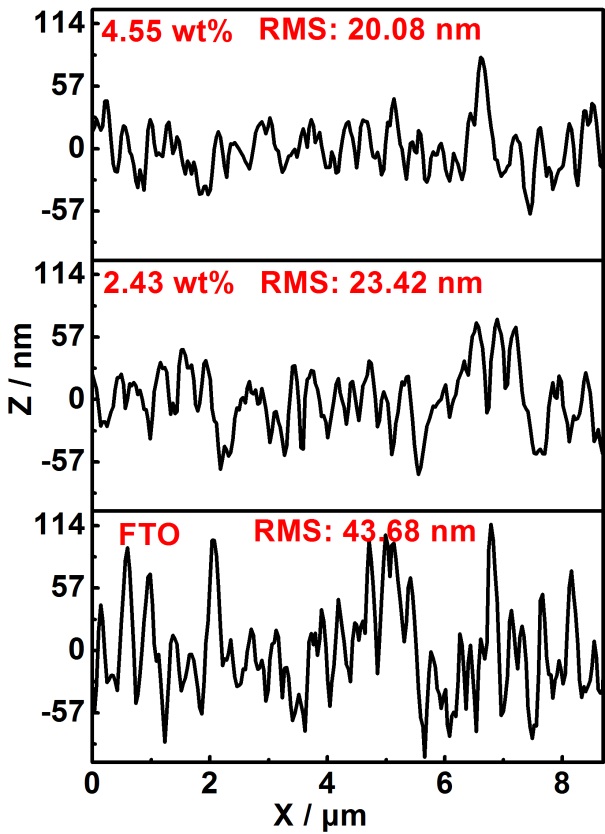


**Figure S8** RMS 2 (see area indicated in Fig. S7) of bare FTO and perovskite films deposited on FTO from precursors with initial concentrations of 2.43 and 4.55 wt%.

**Table S1** Detailed RMS data for each film.

|  | RMS 1 / nm | RMS 2 / nm | RMS 3 / nm | Average RMS / nm |
| --- | --- | --- | --- | --- |
| FTO | 46.72 | 43.68 | 47.33 | 45.91 ± 2.23 |
| 2.43 wt% | 26.75 | 23.42 | 28.89 | 26.35 ± 2.93 |
| 4.55 wt% | 21.77 | 20.08 | 19.92 | 20.59 ± 1.18 |


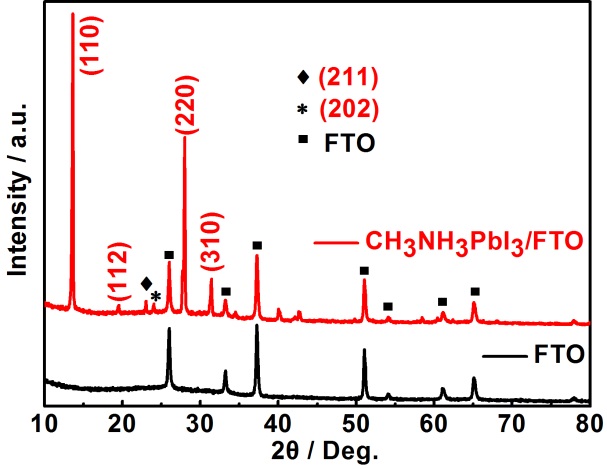


**Figure S9** XRD patterns of the CH_3_NH_3_PbI_3_ film deposited on the FTO substrate and the bare FTO substrate.


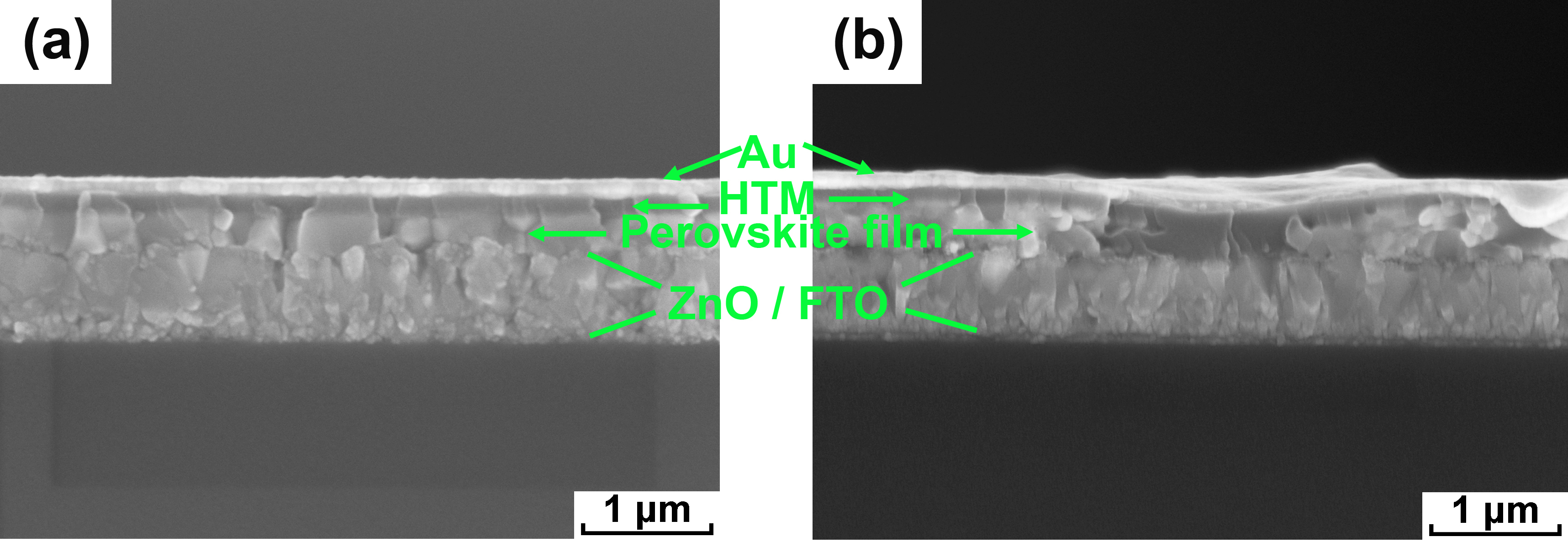


**Figure S10** Cross-sectional views of (a) full-coverage PSC and (b) non-full coverage PSC.

**Table S2** Photovoltaic parameters of PSCs with bare ratio *φ* of ~27%, ~35% and ~67%, where *J*_sc_ is the short circuit density, FF is the fill factor, and *η* is the conversion efficiency.

| Bare ratio *φ* | *J*_sc_ / mA⋅cm^-2^ | *V*_oc_ / V | FF / % | *η* / % |  |
| --- | --- | --- | --- | --- | --- |
| 27% | 5.55 ± 2.50 | 0.654 ± 0.062 | 42 ± 4 | 1.56 ± 0.84 | |
| 35% | 4.02 ± 1.16 | 0.331 ± 0.094 | 38 ± 11 | 0.53 ± 0.25 | |
| 67% | 1.02 ± 0.46 | 0.022 ± 0.014 | 22 ± 9 | 0.005 ± 0.057 | |

**Table S3** Experimental correlation between the bare fraction *φ* and *V*_oc_. All data come from the manuscript and Table S2. ‘Average *φ*’ data are based on *φ*.

| *φ* / % | ~0 | 0-3 | ~3 | 3-6 | ~6 | ~35 | ~67 | ~100 |
| --- | --- | --- | --- | --- | --- | --- | --- | --- |
| Average *φ* / % | 0 | 1.5 | 3 | 4.5 | 6 | 35 | 67 | 100 |
| Average *V*_oc_ / V | 1.2 | 1.14 | 1.04 | 0.98 | 0.8 | 0.33 | 0.02 | 0 |


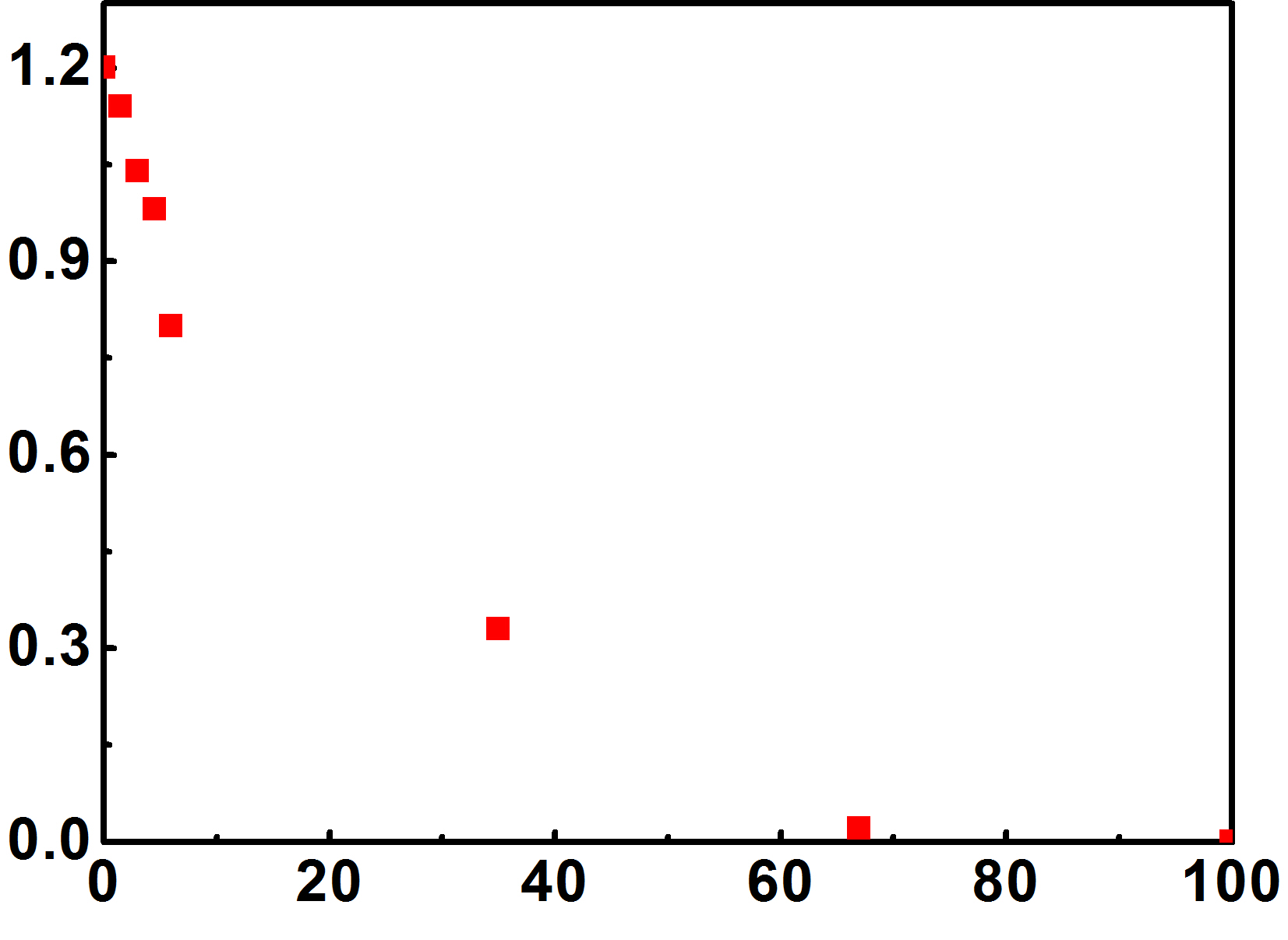


**Figure S11** Correlation between the bare fraction *φ* and *V*_oc_ from Table S3.


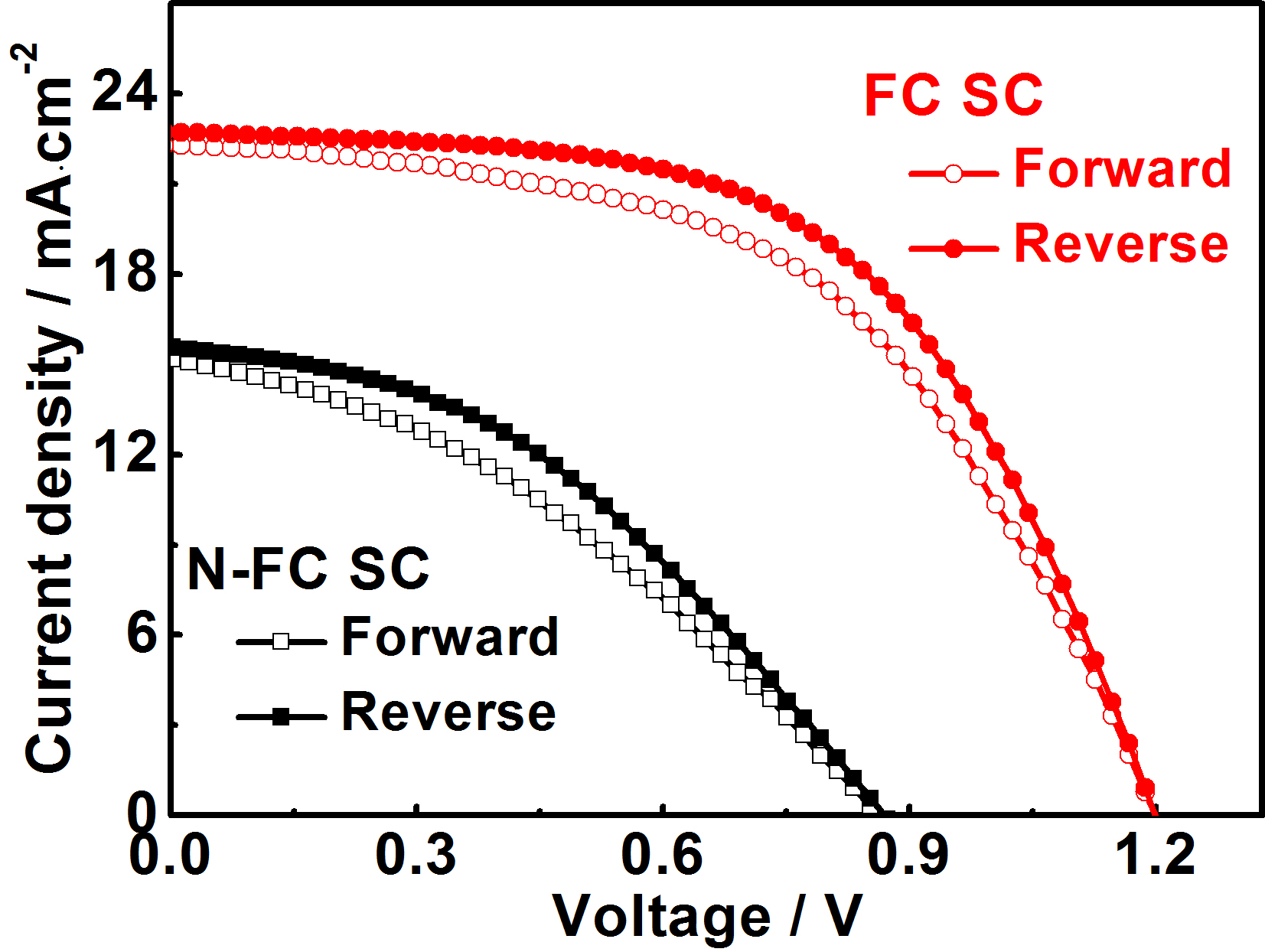


**Figure S12** Typical *J*–*V* curves of the PSCs with *V*_oc_ values of 1.2 and 0.87 V obtained at a scan step of 20.2 mV and delay time of 1,000 ms.

**Table S4 Photovoltaic parameters of the PSCs corresponding to the *J*–*V* curves in Fig. S12.**

| Cell type | *J*_sc_ / mA⋅cm^-2^ | *V*_oc_ / V | FF / % | *η* / % | *R*_sh_ / Ω⋅cm^2^ |
| --- | --- | --- | --- | --- | --- |
| FC SC-Forward | 22.26 | 1.201 | 52.37 | 14.00 | 898.86 |
| FC SC-Reverse | 22.71 | 1.203 | 56.07 | 15.32 | 972.88 |
| N-FC SC-Forward | 15.16 | 0.863 | 33.77 | 4.74 | 323.27 |
| N-FC SC-Reverse | 15.55 | 0.871 | 40.76 | 5.47 | 384.49 |

**Table S5** Detailed photovoltaic parameters of PSCs with *V*_oc_ of 1.2 V.

| Cell No. | *J*_sc_ / mA⋅cm^-2^ | *V*_oc_ / V | FF / % | *η* / % |  |
| --- | --- | --- | --- | --- | --- |
| Cell 1 | 22.74 | 1.203 | 56.02 | 15.32 | |
| Cell 2 | 21.88 | 1.200 | 57.60 | 15.12 | |
| Cell 3 | 22.37 | 1.205 | 55.76 | 15.03 | |
| Cell 4 | 22.33 | 1.202 | 55.42 | 14.87 | |


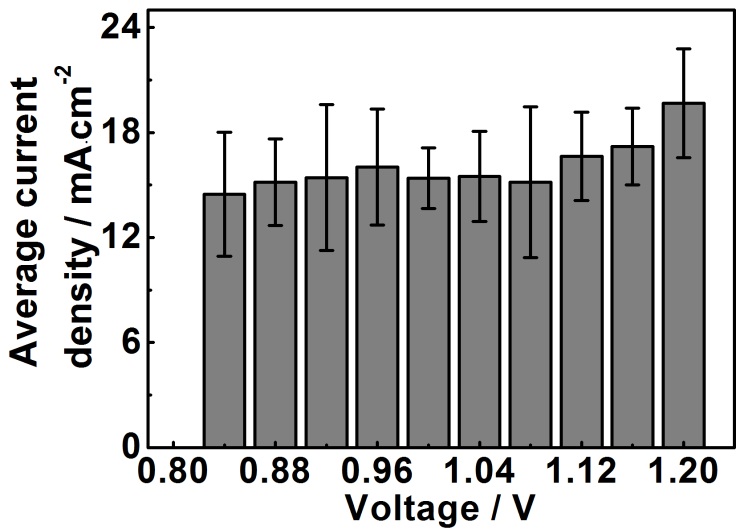


**Figure S13** Average current density and its variation in PSCs 0.275 cm^2^ in size for each *V*_oc_.


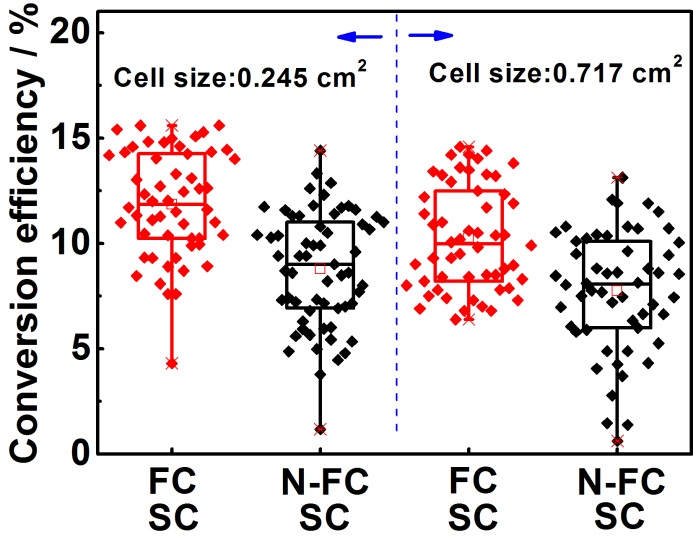


**Figure S14** Conversion efficiency of FC SCs and N-FC SCs with cell sizes of 0.245 and 0.717 cm^2^.

**Detailed statistical calculation of the bare fraction of perovskite films**

A schematic diagram of the calculation process is shown in Fig. S15. First, a perovskite film is viewed at a low magnification of 5,000× so that the incompletely covered areas cannot be clearly distinguished from the perovskite film, and a representative area A of the perovskite film is selected. Second, the selected area A is viewed with a high magnification of 100,000× so that any incompletely covered area appears clearly in the SEM results, and 50 SEM results (B_1_, B_2_, B_3_…..B_50_) in A are selected at random. Third, the bare fraction *φ_n_* of every B*_n_* is calculated using Image J software (Image J2x, 2011). The total bare fraction *φ* can be obtained as follows.

(S23)


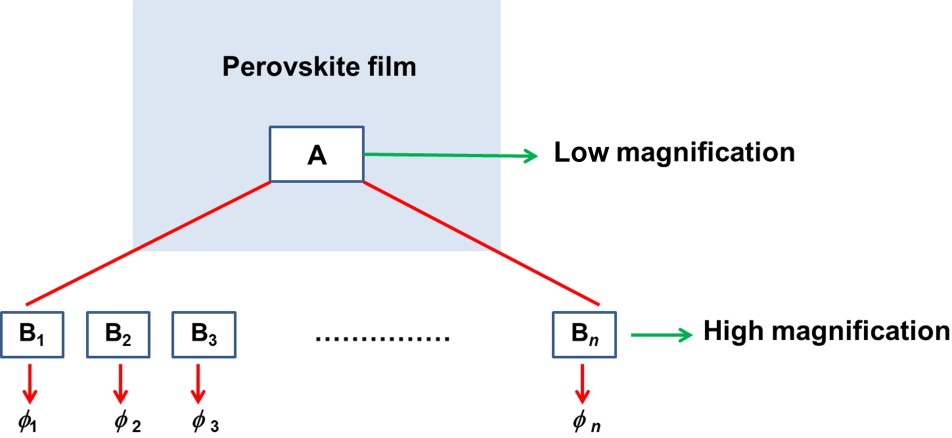


**Figure S15** Schematic diagram of the process for calculating the bare fraction *φ*.

Fig. S16 shows the process for calculating the bare fraction *φ_n_* from B*_n_* using Image J software (Image J2x, 2011). First, the uncovered areas in the SEM image are identified on the basis of their surface morphology. The uncovered area shows a nanoscale rough surface, whereas the surface of the covered area shows large-scale uniformity and flatness. Second, a fill colour is applied in the uncovered areas to help the software to distinguish the uncovered areas using the contrast ratio. Third, the resulting SEM image is imported into the software, and *φ_n_* is exported as the ratio of the uncovered areas filled with colour to the entire area.


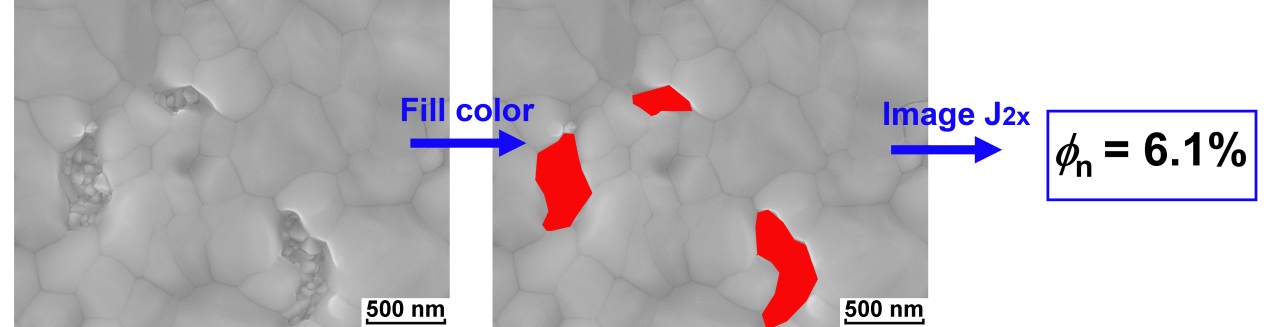


**Figure S16** Process for calculating *φ*_n_ from *B_n_*.
